# Ghrelin Decreases Firing Activity of Gonadotropin-Releasing Hormone (GnRH) Neurons in an Estrous Cycle and Endocannabinoid Signaling Dependent Manner

**DOI:** 10.1371/journal.pone.0078178

**Published:** 2013-10-04

**Authors:** Imre Farkas, Csaba Vastagh, Miklós Sárvári, Zsolt Liposits

**Affiliations:** 1 Laboratory of Endocrine Neurobiology, Institute of Experimental Medicine, Hungarian Academy of Sciences, Budapest, Hungary; 2 Department of Neuroscience, Faculty of Information Technology, Pázmány Péter Catholic University, Budapest, Hungary; University of Cordoba, Spain

## Abstract

The orexigenic peptide, ghrelin is known to influence function of GnRH neurons, however, the direct effects of the hormone upon these neurons have not been explored, yet. The present study was undertaken to reveal expression of growth hormone secretagogue receptor (GHS-R) in GnRH neurons and elucidate the mechanisms of ghrelin actions upon them. Ca^2+^-imaging revealed a ghrelin-triggered increase of the Ca^2+^-content in GT1-7 neurons kept in a steroid-free medium, which was abolished by GHS-R-antagonist JMV2959 (10µM) suggesting direct action of ghrelin. Estradiol (1nM) eliminated the ghrelin-evoked rise of Ca^2+^-content, indicating the estradiol dependency of the process. Expression of GHS-R mRNA was then confirmed in GnRH-GFP neurons of transgenic mice by single cell RT-PCR. Firing rate and burst frequency of GnRH-GFP neurons were lower in metestrous than proestrous mice. Ghrelin (40nM-4μM) administration resulted in a decreased firing rate and burst frequency of GnRH neurons in metestrous, but not in proestrous mice. Ghrelin also decreased the firing rate of GnRH neurons in males. The ghrelin-evoked alterations of the firing parameters were prevented by JMV2959, supporting the receptor-specific actions of ghrelin on GnRH neurons. In metestrous mice, ghrelin decreased the frequency of GABAergic mPSCs in GnRH neurons. Effects of ghrelin were abolished by the cannabinoid receptor type-1 (CB1) antagonist AM251 (1µM) and the intracellularly applied DAG-lipase inhibitor THL (10µM), indicating the involvement of retrograde endocannabinoid signaling. These findings demonstrate that ghrelin exerts direct regulatory effects on GnRH neurons via GHS-R, and modulates the firing of GnRH neurons in an ovarian-cycle and endocannabinoid dependent manner.

## Introduction

The orexigenic peptide, ghrelin was initially discovered in rat stomach [1], where it is secreted into the circulation under certain physiological conditions such as fasting [2-4]. Synthesis of ghrelin was later confirmed in other organ systems, too [5]. It serves as endogenous ligand for the growth hormone secretagogue receptor (GHS-R) [1], which belongs to the G-protein coupled receptor (GPCR) family [6]. Expression of the receptor was detected in the hypothalamo-pituitary unit and several other brain loci [6-9].

The wide-spread expression of ghrelin in various organs shows that it is involved in diverse functions such as growth hormone secretion [10], regulation of food intake and energy balance [2], control of ACTH secretion [11], regulation of insulin and blood glucose levels [12,13], processes of learning and memory, modulation of synaptic plasticity [8,14], and control of reproductive functions [5,15]. Ghrelin production was also revealed in the homeostatic centers of the hypothalamus and the pituitary [1,2,16-18].

Earlier studies have indicated that hypothalamic neurons involved in the central control of the gonadal axis were powerfully affected by ghrelin. Decreased gonadotropin-releasing hormone-1 (GnRH) release was demonstrated from hypothalamic explants after *ex vivo* ghrelin administration in male rats [19]. Furthermore, diminished GnRH secretion by hypothalamic fragments of ovariectomized (OVX) female rats was found following ghrelin application [20]. Kisspeptin mRNA expression was also suppressed by ghrelin in the medial preoptic area (mPOA) [21]. Since GnRH neurons play a pivotal role in the orchestration of the pituitary-gonadal axis, any ghrelin-induced modulation of the GnRH neuronal system has impact on various events of reproductive physiology. In accordance with rodent studies, human data also showed involvement of ghrelin in the inhibition of spontaneous secretion of luteinizing hormone (LH) [22-24]. While the role of ghrelin upon central, hypothalamic regulatory mechanisms of reproduction has been well-established, the way how ghrelin exerts its central effects on the neuronal networks governing reproduction centrally, and particularly on GnRH neurons, has not been fully elucidated, yet.

In the present study, we addressed the putative, direct effects of ghrelin upon GnRH neurons. The performed electrophysiological and molecular biological studies tested the hypothesis that ghrelin acted on GnRH neurons via GHS-R and decreased the firing activity of these cells. Since most of GnRH neurons express ERβ [25,26] and numerous functions of GnRH neurons strongly depend on the estrous-cycle and level of circulating estradiol [27,28], we investigated the effect of ghrelin in GnRH neurons of metestrous and proestrous mice. Change in the firing activity of GnRH neurons correlates with the excitatory GABAergic neurotransmission via GABA_A_-receptors (GABA_A_-R) [29-31], therefore action of ghrelin on the GABAergic input to the GnRH neurons was also studied. In addition, literature data revealed that effect of ghrelin in hypothalamic neurons involved release of endocannabinoid by postsynaptic neurons [32-34], thus the experiments presented here also examined whether modulatory action of ghrelin was manifested via endocannabinoid retrograde signaling to GABAergic neuronal afferents of GnRH neurons utilizing GABA_A_-Rs in neurotransmission.

## Materials and Methods

### Ethics statement

All studies were carried out with permissions from the Animal Welfare Committee of the Institute of Experimental Medicine Hungarian Academy of Sciences (Permission Number: A5769-01) and in accordance with legal requirements of the European Community (Decree86/609/EEC). All animal experimentation described was conducted in accord with accepted standards of humane animal care and all efforts were made to minimize suffering. Sacrifice of animals was carried out by decapitation in deep anesthesia by isoflurane inhalation.

### Cell culture

GnRH-producing immortalized GT1-7 neurons [35] were cultured in Dulbecco Modified Eagle Medium (DMEM, Sigma, St. Louis, MO, US) containing high-glucose and supplemented with 10% fetal calf serum (FCS, Sigma) and 5% horse serum (HS, Sigma). Prior to 17β-estradiol (E2; 1nM, Sigma) treatment, the culturing medium was replaced with a steroid/thyroid- and phenol red-free one and cells were cultured in this medium for 24hrs. Subsequently, one group of the cells was treated with E2 for 24h whereas the other group was cultured further in the steroid-free medium for another 24h. The cells were then processed for calcium imaging experiments.

### Animals

Adult, gonadally intact female and male mice were used from local colonies bred at the Medical Gene Technology Unit of the Institute of Experimental Medicine (IEM). They were housed in light (12:12 light-dark cycle, lights on at 06:00h) - and temperature (22±2°C) controlled environment, with free access to standard food and tap water. GnRH-green-fluorescent protein (GnRH-GFP) transgenic mice (n=70) bred on a C57BL/6J genetic background were used for electrophysiological experiments. In this animal model, a GnRH promoter segment drives selective GFP expression in the majority of GnRH neurons [36]. Phase of the estrous cycle was checked by both evaluating vaginal smear [37] and visual observation of the vaginal opening using the method elaborated recently in the Jackson Laboratory [38].

### Calcium imaging

Cultured GT1-7 neurons were loaded with the calcium-sensitive fluorescent dye Fura-2AM (1µM; Molecular Probes, Eugene, Oregon, US) in loading buffer Hanks’ Balanced Salt Solution (HBSS) containing 0.1% DMSO and Pluronic-F127 (1µM, Molecular Probes) for 1.5h at room temperature. After washing with HBSS, the experiments were carried out in HBSS at room temperature. In each experiment, ghrelin (acylated-ghrelin; 1µM; Tocris, Bristol, UK) was pipetted directly into the bath solution after a 1min baseline recording and then ghrelin remained in the HBSS during recording. The GHS-R antagonist JMV2959 (10µM; Aeterna Zentaris GmBH, Frankfurt, Germany) was added to the HBSS 10min before ghrelin application.

In the case of E2 pre-treatment, the cells were pre-treated with E2 (1nM) as described in the “Cell culture” section and all of rinsing and recording HBSS solutions contained the same concentration of E2, which means that E2 was continuously present in the HBSS during recording.

The experiments were carried out with ARGUS HiSCA Ca^2+^-imaging system (Hamamatsu Photonics, Hamamatsu, Japan). The ratio of the fluorescent signals obtained at excitation wavelengths of 340 and 380nm was used to determine changes in the intracellular Ca^2+^-concentration of the soma of the cells. Ca^2+^-imaging recordings were baseline corrected, then the area-under-curve data of the records representing the net change in the intracellular free Ca^2+^-content were analyzed. Baseline correction was carried out by subtracting the background fluorescence measured from a region in the coverslip free of neurons [39].

### Brain slice preparation and recordings

Mice were deeply anesthetized using Isoflurane inhalation. The brain was removed rapidly and immersed in ice cold sodium-free artificial cerebrospinal fluid (Na-free aCSF) bubbled with a mixture of 95% O_2_ and 5% CO_2_. The solution contained the following (in mM): saccharose 252, KCl 3.5, NaHCO_3_ 26, MgSO_4_ 1.2, NaH_2_PO_4_ 1.25, CaCl_2_ 2.5, glucose 10. Hypothalamic blocks were dissected and 350µm-thick coronal slices (between 1.42-0.6mm measured from the bregma according to Paxinos’ Mouse Brain Atlas [40]) were prepared from the medial septum/preoptic area with a VT-1000S vibratome (Leica) in the ice-cold oxygenated Na-free aCSF. The slices were equilibrated in normal aCSF (in mM): NaCl 135, KCl 3.5, NaHCO_3_ 26, MgSO_4_ 1.2, NaH_2_PO_4_ 1.25, CaCl_2_ 2.5, glucose 10, saturated with O_2_/CO_2_ for 1h. Initial temperature of aCSF was 33°C which was left to cool to room temperature during equilibration.

Male mice and group of metestrous mice were sacrificed between 1100-1200h, whereas proestrous mice and another group of metestrous mice were sacrificed between 1400-1500h.

Recordings were carried out in oxygenated aCSF at 33°C. Axopatch-200B patch-clamp amplifier, Digidata-1322A data acquisition system, and pCLAMP 9.2 software (Molecular Devices Co., Silicon Valley, California, US) were used for recording. Cells were visualized with a BX51WI IR-DIC microscope (Olympus Co., Tokyo, Japan). The patch electrodes (OD=1.5mm, thin wall, Hilgenberg GmBH, Malsfeld, Germany) were pulled with a Flaming-Brown P-97 puller (Sutter Instrument Co., Novato, CA, US) and polished with an MF-830 microforge (Narishige Inc., Tokyo, Japan).

GnRH-GFP neurons were identified by brief illumination at 470nm using an epifluorescent filter set, based on their green fluorescence, typical fusiform shape and topographic location in the preoptic area [36].

Loose-patch or whole-cell patch-clamp measurements were carried out with an initial control recording (4min), then ghrelin (4nM-4μM) was added to the aCSF and the recording continued for a subsequent 11min. When the GHS-R antagonist JMV2959 (10µM), the CB1 antagonist AM251 (1µM; Tocris), the ionotropic glutamate receptor blocker kynurenic acid (2mM) or the GABA_A_-R antagonist picrotoxin (100µM) were used, they were added to the aCSF 10min before starting the recording. Intracellular Ca^2+^-pool was depleted by applying thapsigargin (1µM) in the aCSF for 30min before patch clamping. Each neuron served as its own control when drug effects were evaluated.

### Loose-patch-clamp experiments

Loose-patch measurements were carried out as described earlier [41]. Briefly, pipette potential 0mV, pipette resistance 1-2MΩ, resistance of loose-patch seal 7-40MΩ. The pipette solution contained (in mM): NaCl 150, KCl 3.5, CaCl_2_ 2.5, MgCl_2_ 1.3, HEPES 10, glucose 10 (pH=7.3 with NaOH).

### Whole-cell patch-clamp experiments

The GnRH neurons were measured as described earlier [41]. Briefly, the neurons were voltage-clamped at holding potential -70mV, the intracellular pipette solution contained (in mM): HEPES 10, KCl 140, EGTA 5, CaCl_2_ 0.1, Mg-ATP 4, Na-GTP 0.4 (pH=7.3 with NaOH). The resistance of the patch electrodes was 2-3MΩ. Spike-mediated transmitter release was blocked in all experiments by adding the voltage-sensitive Na-channel inhibitor tetrodotoxin (TTX, 750nM, Tocris) to the aCSF 10min before miniature postsynaptic currents (mPSCs) were recorded. The mPSCs recorded under the circumstances used in our experiments were related to GABA_A_-R activation [41,42].

Current clamp experiments were carried out at 0pA holding current. The aCSF contained ionotropic glutamate receptor blocker kynurenic acid (2mM, Sigma) and GABA_A_-R blocker picrotoxin (100µM, Sigma) to block fast glutamatergic and GABAergic neurotransmission. In order to examine role of L-type voltage gated calcium channels, nifedipine (inhibitor of the L-type channels) was also added to the aCSF (10µM, Sigma). The inihibitors were added to the aCSF 10min before starting recording.

The source of endogenous cannabinoids that regulate GABAergic afferents to GnRH neurons upon ghrelin administration was investigated by adding the diacylglycerol (DAG) lipase inhibitor tetrahydrolipstatin (THL, also known as orlistat; 10µM; Tocris) to the intracellular solution to block 2-arachidonoylglycerol (2-AG) synthesis. To minimize THL spill and therefore any extracellular effect of THL, GnRH cells were approached rapidly (<1min) and the flow rate of aCSF was increased from 5-6 to 8-9 ml/min. Just before release of the positive pressure in the pipette touching the GnRH neuron, the flow rate was restored to 5-6 ml/min to avoid any mechanical movement of the slice. During this short period (<1min) extracellular effect of THL can be considered as negligible, since volume of the recording chamber is small (1.5ml) comparing to the volume of aCSF washing THL out of the chamber. The pipette solution containing THL was allowed to equilibrate with the intracellular milieu of the cell for 20 min before starting recording.

### Harvesting, cDNA synthesis, pre-amplification and real-time PCR of GnRH-GFP neurons

In order to harvest cytoplasm from GnRH-GFP neurons for single-cell PCR experiment, patch pipettes were pulled from capillaries heated previously at 300C for 6h and filled with autoclaved intracellular pipette solution not containing Mg-ATP and Na-GTP. After establishing the whole-cell patch configuration, cytoplasm was harvested by applying gentle negative pressure under visual control [43,44]. Cytoplasmic samples of 10 cells obtained from four male transgenic mice were used.

The harvested cytoplasmic pool was processed for in vitro reverse transcription using SuperScript VILO cDNA Synthesis Kit (Life Technologies, Carlsbad, CA, US). We introduced pre-amplification with 14 cycles for selected targets according to manufacturer’s protocol using TaqMan PreAmp Master Mix (Life Technologies). Low cycle number ensured unbiased amplification and quantitative measurement of gene expression. Subsequently, we performed quantitative real-time PCR on a ViiA7 Real-Time PCR System (Life Technologies) with TaqMan Gene Expression Assays (Life Technologies). Inventoried assays used in both pre-amplification and realtime PCR were as follows: *Gapdh* (assay ID: Mm99999915_g1); *Kiss1r* (Mm00475046_m1); *Gnrh* (Mm01315605_m1); *Ghsr* (Mm00616415_m1). To avoid detection of genomic DNA, each assay was designed to span exons.

### Statistical analysis

Each experimental group included 6-7 animals in the measurements. Recordings were stored and analyzed off-line. Event detection was performed using the Clampfit module of the PClamp9.2 software (Molecular Devices). Bursts were defined according to Lee et al [45]. Mean firing rate was calculated as number of spikes divided by the length of the respective period (4min or 11min). Burst frequency was calculated by dividing number of bursts with the length of the respective time period. Intraburst frequency was calculated by dividing number of spikes with the length of the respective burst. Percentage changes resulted from ghrelin application were calculated by dividing the value to be analyzed before (4min) and after (the subsequent 11 min) ghrelin administration. Instantaneous frequency (i.e. event frequency at the rate of the current and the previous event) was calculated using the Clampfit module of the PClamp software.

Group data were expressed as mean±standard error (SEM). Statistical significance was analyzed using the Student’s t-test or ANOVA followed by Newman-Keuls post-hoc test (GraphPad Software Inc., La Jolla, California, US), and considered at p<0.05 (i.e. 95% confidence interval).

## Results

### Ghrelin increased the intracellular free Ca^2+^-content in GT1-7 neurons

**Figure 1:**
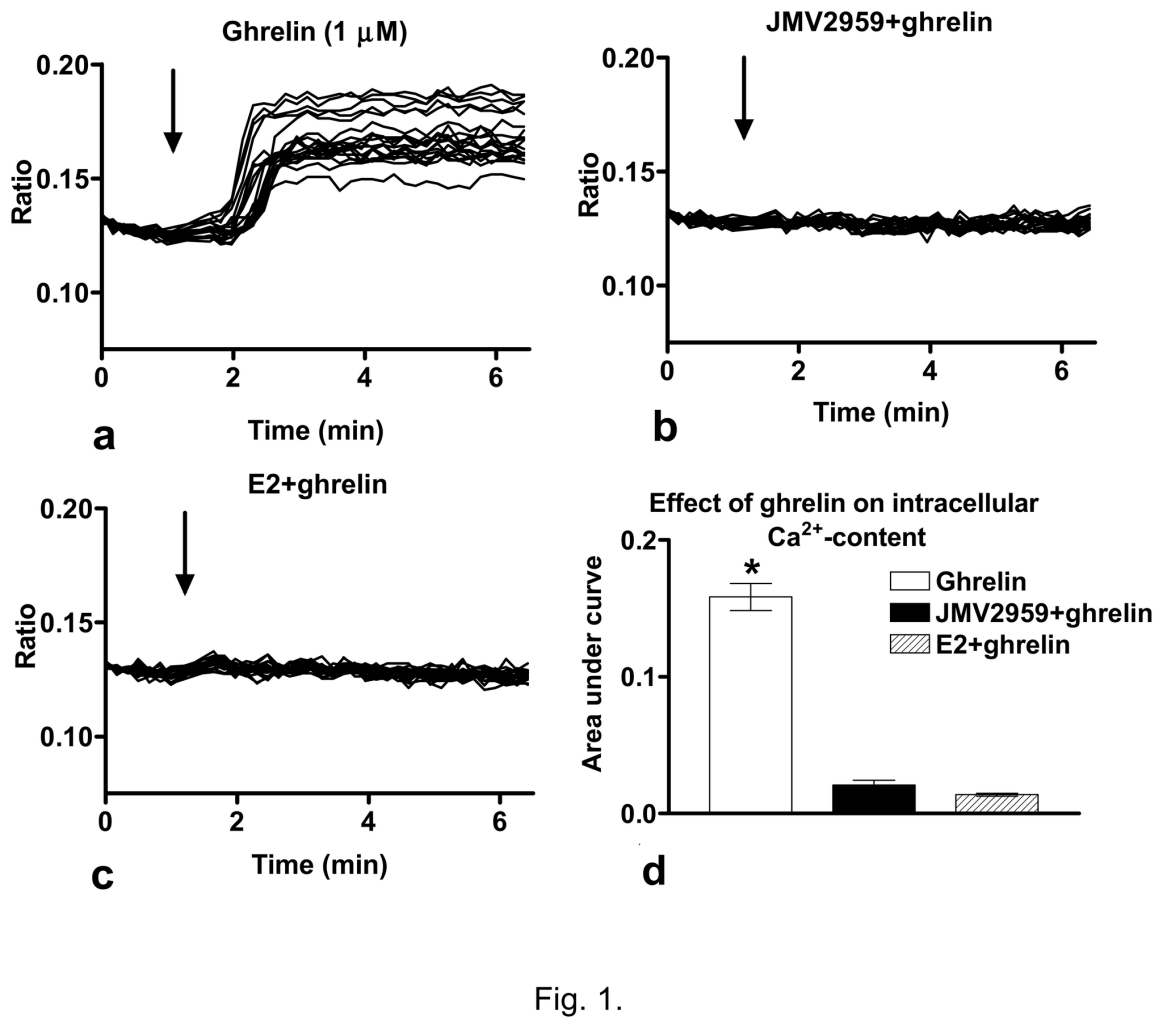
Effect of ghrelin on the intracellular free Ca^2+^-content of the GT1-7 neurons. **a**) Ghrelin (1 µM) administration in an estrogen-free medium resulted in an increase in the intracellular Ca^2+^-content. **b**) The GHS-R antagonist JMV2959 (10 µM, 10 min) abolished the action of ghrelin. **c**) Estradiol (E2, 1 nM, 24 h) treatment eliminated the effect of ghrelin on the intracellular Ca^2+^-concentration. **d**) Bar graph of the area-under-curve data representing the net change in the free Ca^2+^-content. *=p<0.0001. Arrow shows the onset of ghrelin administration.

In order to test effect of ghrelin in GT1-7 neurons, the hormone was applied into the bath of the cells cultured in steroid-free medium, and the change in the intracellular free Ca^2+^-content was measured. Ca^2+^-imaging demonstrated that ghrelin (1µM) evoked a robust increase in the intracellular Ca^2+^-content which started in less than 1min (36 cells in three repeated experiments; Figure **1a**). Pretreatment of the cells with the GHS-R antagonist JMV2959 (10µM, 10min) eliminated the ghrelin-evoked elevation of the intracellular free Ca^2+^-concentration suggesting involvement of the GHS-R (36 cells in three repeated experiments; Figure **1b**). The ghrelin-triggered rise in the intracellular Ca^2+^-content was also abolished by applying 17β-estradiol (E2, 1nM, 24h) in the steroid-free medium indicating an interaction between the ghrelin and E2 signaling mechanisms in GT1-7 neurons (36 cells in three repeated experiments; Figure **1c**). The area-under-curve data representing the net change in the intracellular free Ca^2+^-concentration showed a significantly higher value when ghrelin was added alone than the one obtained after JMV2959 or E2 pretreatment (Figure 1d; p<0.0001).

### GnRH neurons expressed Ghsr mRNA

**Figure 2:**
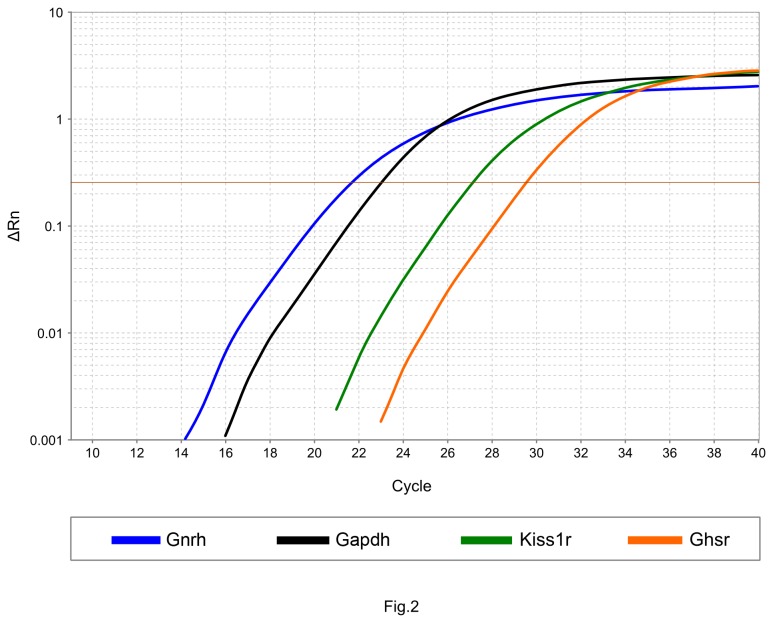
Expression of GHS-R mRNA in GnRH-GFP neurons. Expression of GHS-R mRNA in GnRH-GFP neurons was demonstrated by single cell real-time PCR. The amplification plot shows the expression of *Gnrh* (Ct: 20.4), *Kiss1r* (Ct: 27) and *Gapdh* (Ct: 22.4) genes. The regular amplification curve of *Ghsr* with Ct values of 29.6 proves the expression of GHS-R mRNA.

We employed GnRH-GFP transgenic mice, in which the green fluorescent neurons were clearly distinguishable in the medial septum and the mPOA. Individual GFP-expressing GnRH neuronal cells were identified in the brain slices and then their cytoplasmic content was harvested by patch pipettes. To validate GnRH neurons, the expression of two characteristic markers, GnRH and kisspeptin1 receptor mRNAs (*Gnrh* and *Kiss1r*) was monitored. Expression of both *Gnrh* and *Kiss1r* mRNAs was verified in GnRH-GFP samples at cycle threshold (Ct) values of 20.4 and 27, respectively. Amplification curve shows that GnRH neurons also expressed GHS-R mRNA (*Ghsr*) at Ct 29.6 (pool of 10 cells from 3 animals, Figure **2**). Two controls were applied, a non-template control and a non-GFP cell control (pool of 10 GFP-negative neurons chosen from the close vicinity of the GFP-positive neurons from 3 animals). The measurements demonstrated no expression of *Ghsr* mRNA in these controls (not shown).

### Firing characteristics of GnRH neurons of the female and male mice

**Figure 3:**
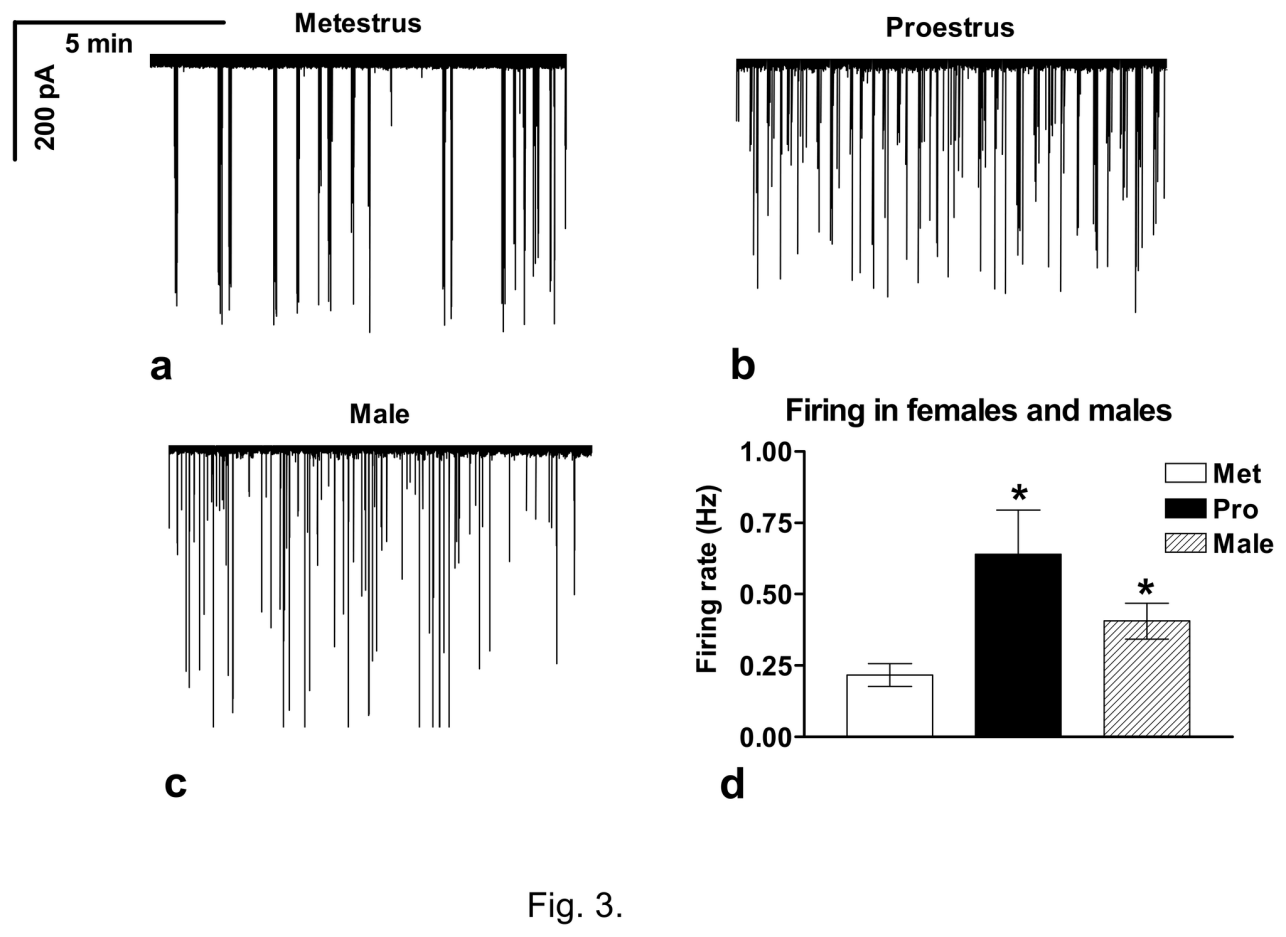
Recordings of firing of GnRH neurons in the brain slice of the female (a,b) and male (c) mice. **d**) Firing rate of GnRH neurons is higher in proestrous than in metestrous female and male mice. Met=metestrus; Pro=proestrus; *=p<0.05.

Loose-patch recordings of the GnRH-GFP neurons of the mouse brain slices showed burst firing both in female and male mice (Figure **3a-c**). Nevertheless, both mean firing rate and burst frequency were significantly lower in metestrous (mean firing rate=0.21±0.04Hz; burst frequency=0.027±0.008Hz, Figure **3a**) than in proestrous mice (mean firing rate=0.64±0.15Hz; burst frequency=0.119±0.031Hz; Figure **3b**) (p<0.05 for both mean firing rate and burst frequency). Both mean firing rate and burst frequency were higher in male mice (mean firing rate=0.40±0.06Hz; burst frequency=0.08±0.026Hz; Figure **3c**) than in metestrous, but lower than in proestrous mice.

Bar graph presents the mean firing rate values of GnRH neurons in the female and male mice (Figure **3d**) and demonstrates that mean firing rate was significantly higher in both proestrous female (16 cells from 5 animals) and male (14 cells from 5 animals) than in the metestrous female (18 cells from 6 animals) mice (p<0.05). The basic burst parameters such as burst frequency, events-in-burst, and intraburst frequency are indicated in Table **1**. The burst frequency value was significantly higher in the proestrous female and male than in metestrous female mice (p<0.05), whereas events-in-burst and intraburst frequency showed no difference.

**Table 1:** Basic burst parameters (before ghrelin application) and the percentage they changed to (after ghrelin administration).

	**Basic burst parameters**			**Changes resulted from ghrelin**		
	**bf (Hz)**	**eb (events)**	**ibf (Hz)**	**bf %**	**eb %**	**ibf %**
**Metestrus**	0.027±0.008	2.50±0.606	6.54±2.219	61.8±14.87*	134.3±28.84	119±28.8
**Proestrus**	0.119±0.031*	3.78±0.737	6.42±2.972	93.7±8.04	123.6±25.82	107.7±5.8
**Male**	0.080±0.026*	2.80±0.329	6.06±0.855	68.5±9.43*	139.6±44.75	122±15.7

bf=burst frequency; eb=events-in-burst; ibf=intraburst frequency. *=p<0.05

### Ghrelin decreased firing of GnRH neurons in metestrous and male but not in proestrous mice

**Figure 4:**
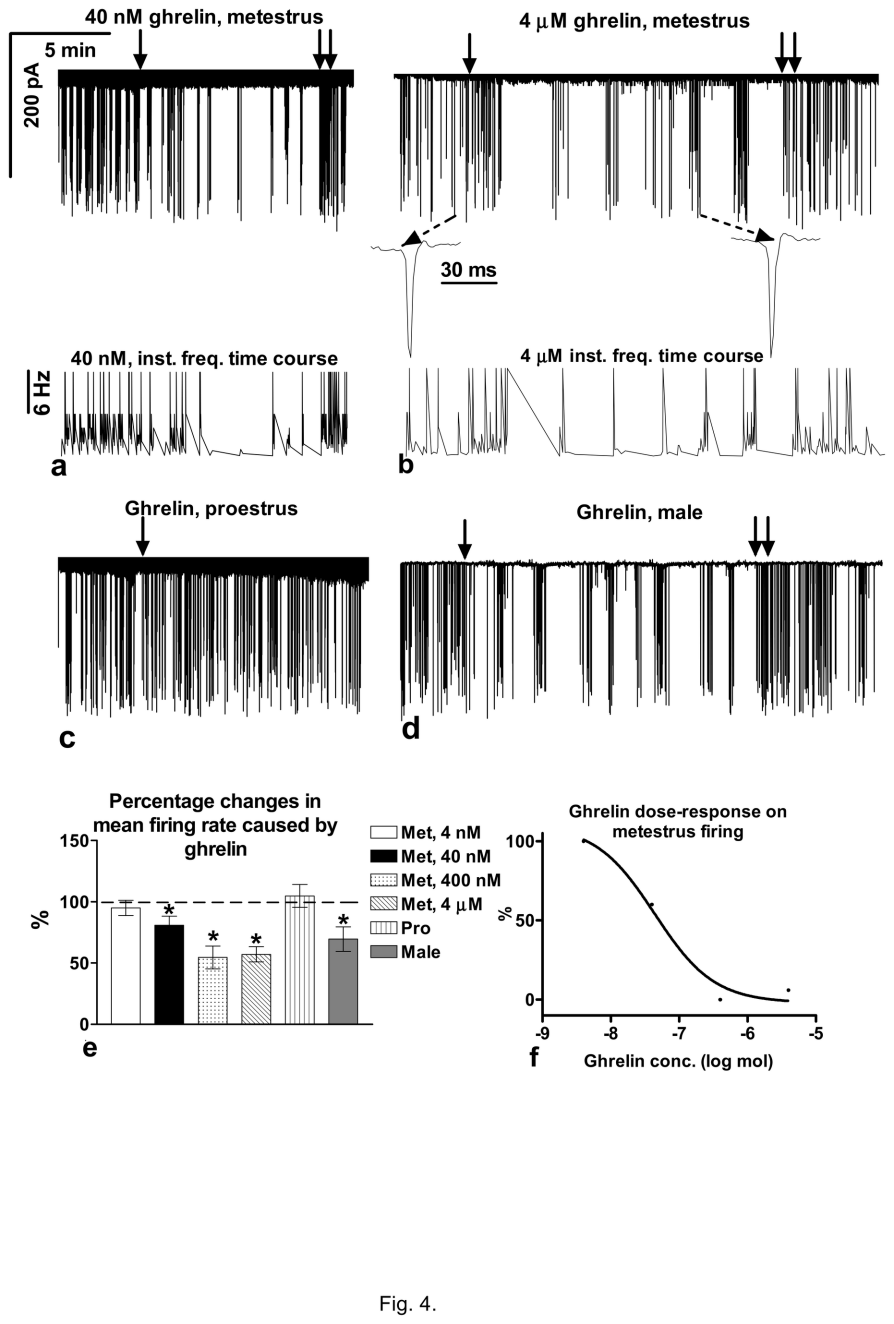
Effect of ghrelin on the firing of the GnRH neurons from the brain slice of the female (metestrus and proestrus) and male mice. **a** and **b**) Ghrelin (40 nM N=4 and 4 µM N=14) decreased the firing rate in the metestrus with no change in the shape of the individual spikes (insets). Time course of the instantaneous frequency is figured under the firing recordings. **c**) In proestrus, firing rate showed no change (N=11). **d**) Ghrelin administration in the male mouse resulted in a decrease in the firing rate (N=15). **e**) Bar graph shows the significant changes in the firing rate in the metestrous female (40nM-4 µM but not at 4 nM) and male, whereas ghrelin exerted no effect in the proestrous female mice. **f**) Dose-response curve of effect of ghrelin on the firing in metestrous mice (0%=full effect, 100%=no effect). Met=metestrus; Pro=proestrus. Arrow shows onset of ghrelin administration whereas double arrow marks wash-out. *=p<0.05.

Ghrelin (4nM) administration in GnRH neurons of the metestrous mice revealed no change in any of the firing parameters measured (mean firing rate from 0.2±0.03 to 0.19±0.02Hz, instantaneous frequency from 6.0±0.4 to 5.8±0.7Hz in 4 neurons from 2 animals, not shown). Higher concentration of ghrelin (40nM) diminished mean firing rate from 0.21±0.02 to 0.17±0.02Hz (80.9±7.4%), and instantaneous frequency from 5.9±0.6 to 4.7±0.5Hz, duration (period from the point when ghrelin started to act till its effect vanished) was 8.5±2.6min in 4 of 5 neurons (from 2 animals) (Figure 4a, p<0.05). 400nM ghrelin decreased mean firing rate significantly from 0.22±0.03 to 0.12±0.03Hz (54.54±9.34%) and instantaneous frequency from 6.4±0.6 to 4.5±0.4Hz (p<0.05) and duration was 21.2±4.1min in 13 of 15 neurons (from 4 animals, not shown). 4µM ghrelin reduced mean firing rate from 0.21±0.03 to 0.12±0.02Hz (57.1±6.2%), instantaneous frequency from 6.09±0.33 to 4.2±0.44Hz, and burst frequency from 0.027±0.008 to 0.016±0.006Hz (61.8±14.87%) significantly with duration of 22.6±3.4min in 12 of 14 neurons (from 5 animals) (Figure 4b; p<0.05). Second ghrelin administration (the same concentration as the first one) applied to the same neuron 5min after wash-out exerted no effect. Table **1** shows that ghrelin application exerted no significant change in the events-in-burst (2.5±0.606 events) and intraburst frequency (6.54±2.22Hz) parameters. Amplitude and shape of the action currents showed no change upon ghrelin treatment at any concentration applied (insets at 4µM in Figure **4b**). When the effect of ghrelin (4µM) on the firing of GnRH neurons of the proestrous mice was investigated, no significant alteration could be detected in any of the firing parameters (in 11 neurons from 4 animals), mean firing rate changed from 0.64±0.15 to 0.67±0.12Hz (104.8±9.41%) (Figure **4c**), instantaneous frequency from 10.2±0.8 to 10.6±0.7Hz, burst frequency from 0.119±0.031 to 0.111±0.028Hz (93.7±8.04%), events-in-burst from 3.788±0.737 to 4.649±0.821 events (123.6±25.8%), and intraburst frequency from 6.42±2.97 to 6.87±2.85Hz (107.7±5.8%) (Table **1**), suggesting that the ability of ghrelin to influence firing activity of GnRH neurons was estrous cycle-dependent. Ghrelin (4µM) administration resulted in significantly reduced firing activity in male mice in 12 of 15 neurons (from 4 animals), mean firing rate changed from 0.40±0.04 to 0.24±0.03Hz (60.5±5.82%), instantaneous frequency from 8.0±0.6 to 6.2±0.5Hz, and burst frequency from 0.08±0.026 to 0.054±0.028Hz (68.5±9.43%) (Figure 4d; p<0.05) with duration of 20.4±3.7min, whereas amplitude, events-in-burst and intraburst frequency parameters were not influenced (Table **1**). Bar graph shows the percentage changes in the mean firing rate resulted from ghrelin application (Figure **4e**), demonstrating that ghrelin significantly decreased mean firing rate in the metestrous female and male (p<0.05), but not in the proestrous female mice. Dose-response curve of ghrelin effect on mean firing rate in metestrous mice showed that EC50 was 52.2nM (Figure **4f**). When metestrous mice were sacrificed in the afternoon, ghrelin (4µM) decreased mean firing rate to 57.6±6.2% (from 0.21±0.02 to 0.12±0.03Hz) to an extent similar to the value found in the experiments performed in the morning (not shown). Instantaneous frequency of firing also diminished (from 5.97±0.47 to 4.8±0.31Hz) in 4 of 5 neurons (from 3 animals), and duration was 21.7±2.8min. The results, therefore, revealed no circadian time dependency of the effect of ghrelin.

**Figure 5:**
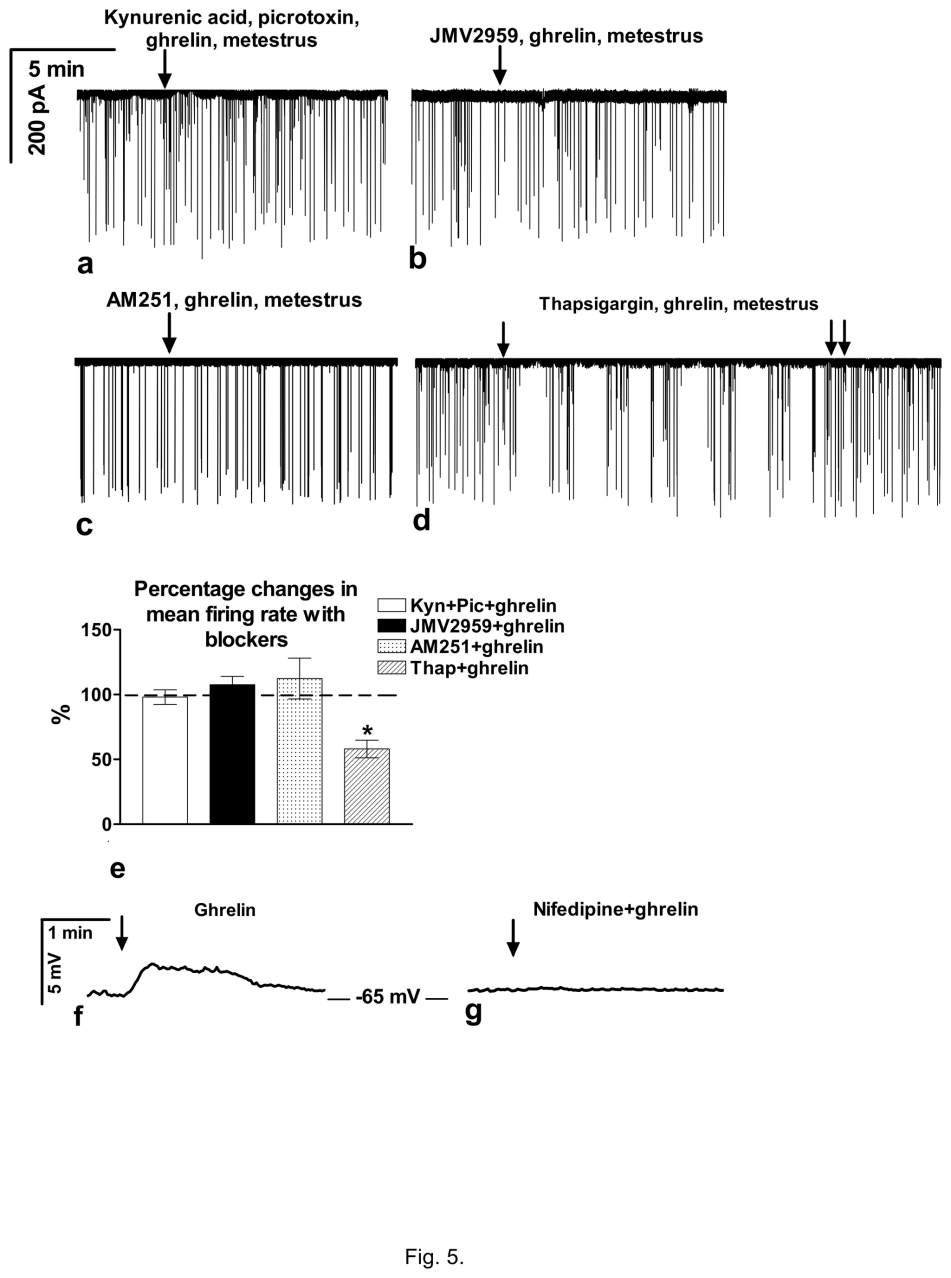
Effect of antagonists on the ghrelin-modulated firing activity and resting potential of GnRH neurons in metestrous mice. **a**) Block of fast neurotransmission by kynurenic acid (kyn) and picrotoxin (pic) eliminated the action of ghrelin (N=4). **b**) The GHS-R antagonist JMV2959 also abolished effect of ghrelin (N=11). **c**) Antagonizing the endocannabinoid CB1 receptor by AM251 abolished effect of ghrelin (N=10). **d**) Depletion of the intracellular Ca^2+^-pools by thapsigargin (thap) showed no effect on the ghrelin-induced decrease in the firing activity (N=4). **e**) Bar graph shows elimination of effect of ghrelin by inhibition of fast neurotransmission, by antagonizing ghrelin receptor or by block of CB1 but not by depleting the intracellular Ca^2+^-sources. **f**) Current clamp measurement showed slight depolarization upon ghrelin administration. **g**) Depolarizing effect of ghrelin was eliminated by nifedipine. *=p<0.05.

Simultaneous blockade of Glu-Rs and GABA_A_-Rs eliminated the effect of ghrelin (mean firing rate from 0.22±0.02 to 0.23±0.02Hz, instantaneous frequency from 6.05±0.37 to 6.12±0.33Hz), showing involvement of fast neurotransmission in its action in 4 neurons (from 2 animals) (Figure **5a**). Pretreatment of GnRH neurons of brain slice of metestrous mice with JMV2959 (10µM, 10min) inhibited the effect of ghrelin (4µM) (in 11 neurons). The mean firing rate did not change significantly (from 0.2±0.03 to 0.2±0.03Hz (107.5±6.41%) and instantaneous frequency from 6.11±0.34 to 6.0±0.29Hz) (Figure **5b**), demonstrating involvement of GHS-R. JMV2959 alone had no effect on the firing parameters. The observed effect of ghrelin (4µM) was also abolished by treatment of the slice with the endocannabinoid receptor type-1 (CB1) antagonist AM251 (1µM, 10min, 10 neurons, 3 animals). The mean firing rate did not alter significantly (from 0.25±0.02 to 0.24±0.03Hz) (Figure **5c**) and the instantaneous frequency (from 7.05±0.46 to 7.0±0.39Hz) suggesting that endocannabinoid signaling mechanism was implied in the process. In order to determine source of Ca^2+^ in the effect of ghrelin, thapsigargin (1µM, 30min) was applied to deplete intracellular Ca^2+^ pools. Thapsigargin was unable to influence effect of 4µM ghrelin (mean firing rate changed to 61.9±5.7% from 0.21±0.03 to 0.13±0.03Hz, instantaneous frequency from 6.1±0.30 to 4.2±0.41Hz, duration was 22.4±3.6min, p<0.05 in 4 of 4 neurons, 2 animals) (Figure **5d**). Bar graph shows that no significant changes in the mean firing rate could be observed when ghrelin was co-applied with JMV2959, the AM251, kynurenic acid and picrotoxin, whereas thapsigargin was ineffective (Figure **5e**). Current clamp measurement revealed that ghrelin (4 µM) triggered slight depolarization (by 3.2±0.6mV from -65.4±0.9mV) in GnRH neurons in the presence of TTX, kynurenic acid and picrotoxin suggesting direct effect of ghrelin in these neurons (Figure **5f**). This effect was eliminated by nifedipine administration (Figure **5g**) showing role of L-type voltage gated calcium channels in this action.

### Effect of ghrelin on the GABAergic mPSCs of GnRH neurons in metestrous mice

**Figure 6:**
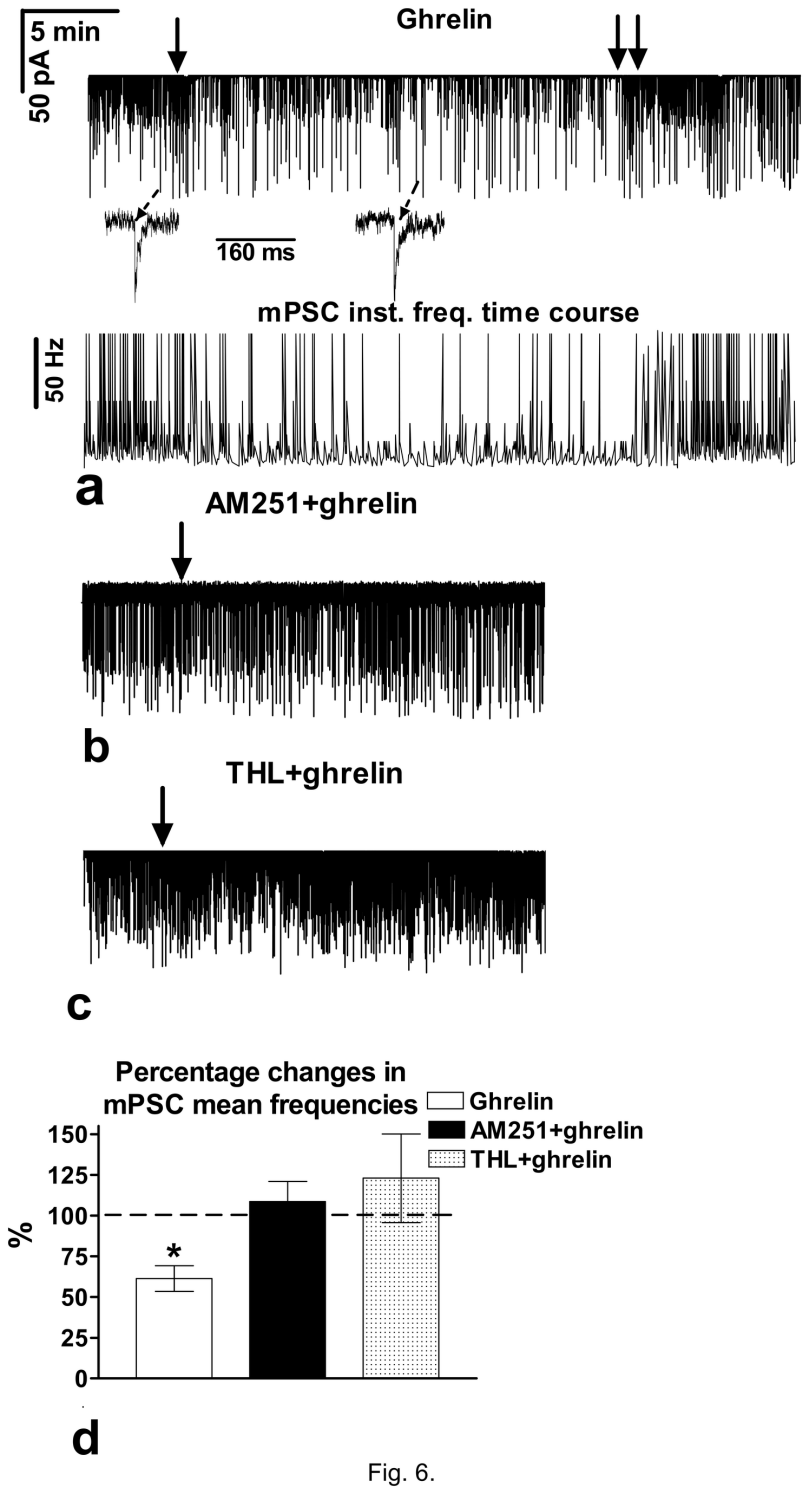
Effect of ghrelin (4 µM) on the mPSCs in the GnRH neurons of the female metestrous mice. **a**) Ghrelin decreased the frequency of the mPSCs (N=14) with no change in the shape of the individual mPSCs (insets). Time course of instantaneous frequency is depicted under the mPSC-recording. **b**) Effect of ghrelin on the mPSCs was abolished by the pretreatment with AM251 (N=11). **c**) Intracellularly applied THL eliminated the ghrelin-evoked changes on the mPSCs (N=10). **d**) Bar graph reveals that ghrelin significantly diminished the frequency of mPSCs. Arrow shows the onset of ghrelin administration. *=p<0.05.

In order to demonstrate the direct action of ghrelin on GnRH neurons of brain slice from metestrous mice, GABAergic miniature postsynaptic currents (mPSCs) were recorded using whole cell patch clamp method, and with 750nM tetrodotoxin (TTX) in the aCSF to block voltage-gated sodium channels and thus, propagation of the action potentials. Administration of ghrelin (4µM) resulted in a significant decrease in the mean frequency of the mPSCs in GnRH neurons (Figures **6a**, 12 of 14 neurons from 4 animals, p<0.05), it changed to 58.4±6.72% of the values before ghrelin application (from 1.11±0.256 to 0.64±0.151Hz). Mean instantaneous frequency also diminished (from 34.6±5.32 to 20.4±4.48Hz). Amplitude of the mPSCs, however, showed no alteration (from 32.7±2.40 to 30.90±2.91pA). Rise and decay time constants of the individual mPSCs revealed no modification, either (τ_r_ from 6.42±1.54 to 7.05±1.72ms; whereas τ_d_ from 28.7±1.96 to 27.2±1.99ms) as can be seen in the insets of Figure **6a**. Time course of the instantaneous frequency of the mPSCs also showed the ghrelin-induced alteration (Figure **6a**). These results demonstrated a presynaptic modulation of the GABA-drive by ghrelin. Pretreatment of the slices with AM251 (1µM, 10min) eliminated the action of ghrelin on the mean frequency of mPSCs in 11 neurons (from 1.58±0.20 to 1.70±0.33Hz, 108±12.24%, 4 animals) and instantaneous frequency (from 44.3±6.2 to 47.8±5.8Hz) (Figure **6b**), suggesting involvement of the endocannabinoid system. Implication of a retrograde endocannabinoid signaling mechanism was confirmed by intracellular application of the DAG-lipase inhibitor THL (10µM) which also abolished the effect of ghrelin on the mean frequency of mPSCs in the 10 neurons (3 animals) measured (from 1.48±0.28 to 1.80±0.37Hz) and instantaneous frequency (from 40.3±7.44 to 48.5±8.1Hz) (Figure **6c**), revealing a direct effect of the hormone in the GnRH neurons. Bar graph summarizes the effect of ghrelin on the mean frequency of the mPSCs and inhibition of the ghrelin-triggered action by antagonizing CB1 and blocking 2-AG synthesis (Figure **6d**).

## Discussion

Previous studies described indirect ghrelin effects on GnRH neurons via modulation of kisspeptin neurons [46,47]. The present study provides evidence for direct action of ghrelin on GnRH secreting neurons. Accordingly, 1) GHS-R mediated the effect of ghrelin in GnRH neurons; 2) ghrelin affected GnRH neurons in an estrous-cycle dependent manner; 3) action of ghrelin involved activation of a retrograde endocannabinoid signaling mechanism targeting GABAergic neurotransmission.

### GHS-R mediated the effect of ghrelin in GnRH neurons

The first evidence for the direct action of ghrelin came from our calcium imaging measurements in GT1-7 neurons, where a robust increase in the intracellular free Ca^2+^-level was recorded upon ghrelin administration. Similar rise in the Ca^2+^-content after ghrelin administration has recently been observed in GT1-7 neurons [48]. In addition, our results showed inhibition of the ghrelin-evoked elevation of the Ca^2+^-concentration by the GHS-R antagonist JMV2959 confirming the presence of functional and active GHS-Rs in GT1-7 neurons. The proof for mRNA expression of Ghsr in GnRH neurons of GnRH-GFP transgenic mice further supported the presence of GHS-R in the GnRH-producing neurons. The expression of ghrelin receptor suggested that binding of ghrelin to its receptor could evoke direct, specific effect in GnRH-producing neurons.

### Ghrelin affects GnRH neurons in an estrous-cycle dependent manner

**Figure 7:**
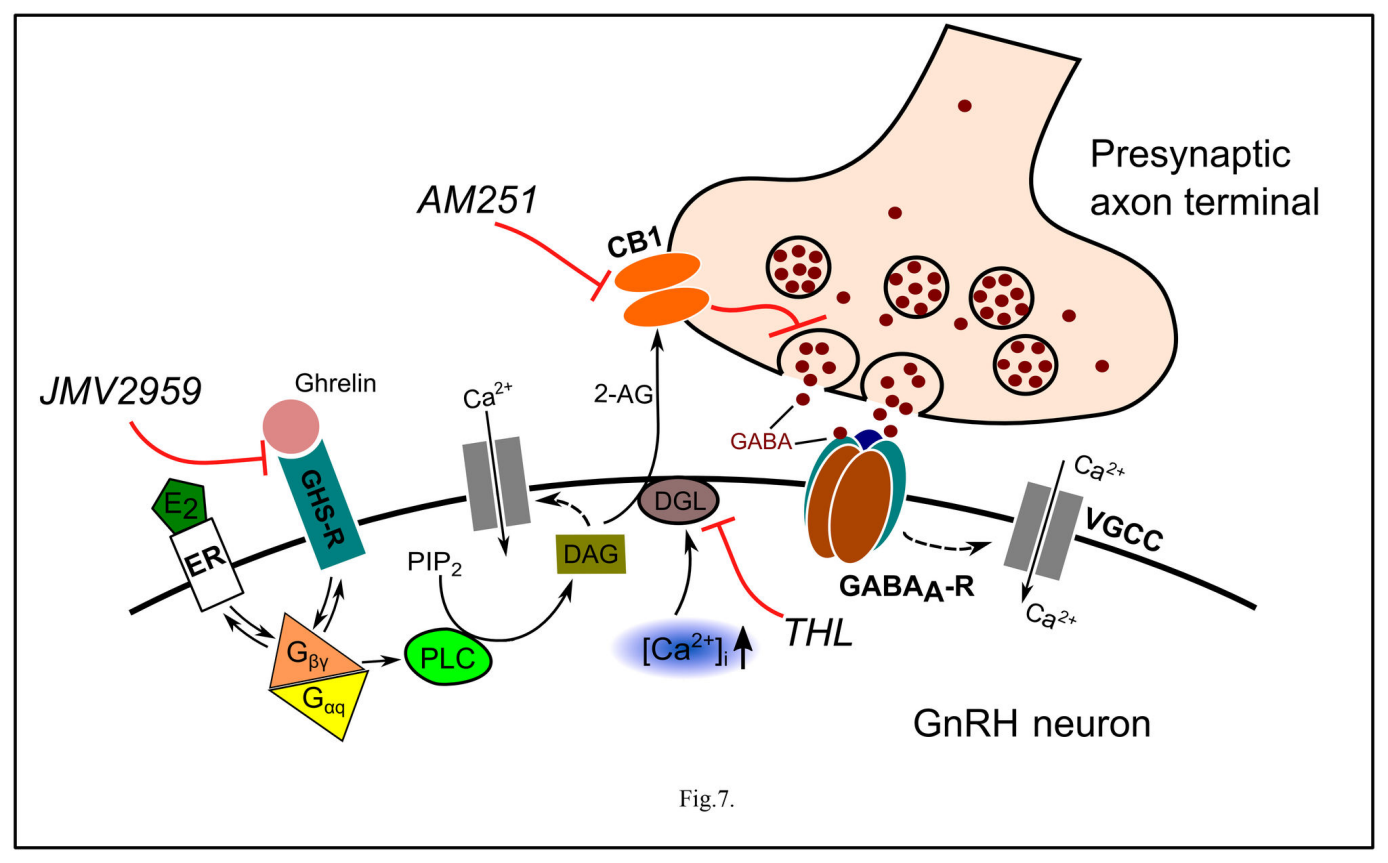
Schematic illustration of E2-dependent effects of ghrelin in GnRH neuron. E2: 17β-estradiol; ER: estrogen receptor; GHS-R: ghrelin receptor; G_βγ_ and G_αq_: G-protein subunits; DAG: diacylglycerol; DGL: DAG-lipase; CB1: cannabinoid receptor type-1; AM251: CB1 antagonist; 2-AG: 2-arachidonoylglycerol; THL: tetrahydrolipstatin (DAG-lipase inhibitor); PIP_2_: phosphatidylinositol 4,5-bisphosphate; PLC: phospholipase-C; GABA_A_-R: GABA_A_ receptor; [Ca^2+^]_i_: intracellular free calcium; VGCC: voltage-gated calcium channel; JMV2959: GHS-R antagonist. Dashed arrow denotes putative indirect action. Binding of ghrelin to GHS-R increases intracellular free Ca^2+^-content E2-dependently, that in turn activates synthesis and release of 2-AG in the postsynaptic GnRH neuron. The released endocannabinoid then binds to the CB1 located in the presynaptic terminal and eventually causes suppression of GABA release into the synaptic cleft.

The study has also revealed that pretreatment of GT1-7 neurons with E2 abolished the ghrelin-triggered increase in the Ca^2+^-content, suggesting an interaction between the ghrelin-activated and the E2-modulated signal transduction pathways. This finding has been supported by results demonstrating that male and OVX female rats were significantly more sensitive than intact female rats to the orexigenic effects of both centrally and systemically administered ghrelin [49]. Similar interaction was concluded by other researchers presenting reduced anorexigenic effects of a GHS-R antagonist treatment in mice after estrogen supplementation [50]. These findings indicate a convergence of the two signaling systems and an increased regulatory potential of ghrelin at low E2 milieu. Nevertheless, the intriguing question is the common point(s) of the two pathways. Our present result that E2 inhibited the ghrelin-evoked rise in the Ca^2+^-content in GT1-7 neurons suggested convergence elements of the pathways upstream to the Ca^2+^-sources. The pathways coupled to GHS-R and the membrane-associated estrogen receptor (ER) could explain the interaction, as proposed in Figure **7**. Both of GHS-R and membrane-associated ER are coupled to G-proteins, and the ghrelin/E2-activated pathways involve phosphatidylinositol 4,5-bisphosphate (PIP_2_), phospholipase-C (PLC), and the voltage-gated Ca^2+^-channels (Figure **7**) [6,51-57]. Nevertheless, this concept requires further elaboration.

The loose patch clamp electrophysiology measurements demonstrated burst-type firing in most of the GnRH neurons recorded, a pattern characteristic for GnRH neurons in both males and females [45,58-60]. However, firing rate and burst frequency in GnRH neurons were significantly higher in proestrous than metestrous mice. Estrous-cycle dependency of the firing rate of the GnRH neurons has already been suggested in a mouse model, where firing parameters in OVX and E2 supplemented mice were compared [59,61-63]. In this model, higher firing rate was observed during positive E2 feedback than under negative feedback, correlating with the proestrus-afternoon and metestrus phases of the estrous-cycle, respectively. Extracellular recordings in the mediobasal hypothalamus also showed volleys of firing preceding LH pulses in ewes and rats [64-66]. Interestingly, in a GnRH-Pericam transgenic mouse model [67] where the intracellular Ca^2+^-content can be monitored real-time in individual GnRH neurons of the acute brain slice, the ratio of the GnRH neurons bursting with high/low frequency did not change throughout the estrous cycle [55]. Another study reported decreased electrical activity in GnRH neurons of Pericam mice in proestrus (compared to diestrus) and in adult (compared to prepubertal) by measuring the calcium transients in these neurons [60]. This is in contrast with other reports demonstrating increased electrical activity of GnRH neurons in proestrus [64-66,68-70]. Discrepancy could be due to the different temperature during recording, slice thickness, flow rate of the extracellular solution, O_2_/CO_2_ bubbling, composition of aCSF, the different genetic design of the transgenic mouse strain and many other details of the experimental design. In addition, the results with the Pericam mice emphasizes that the E2 feedback in the GnRH neurons of the mouse brain is likely to be multimodal in nature with differing mechanistic and temporal domains for each mode and the acute actions of E2 may be one contributor to the feedback mechanism [71].

Our loose-patch recordings revealed a decreased firing and bursting activity of the GnRH neurons of both the metestrous and male mice due to the ghrelin administration. The result is supported by the *ex vivo* observation showing a reduction in the GnRH pulse frequency resulted from ghrelin administration in male rats [19]. The GnRH-release measured in hypothalamic fragments in OVX rats was also suppressed upon *in vitro* ghrelin application [20]. Effect of ghrelin on the reproductive axis, however, is rather complex because it highly depends on the site of action. Acting at the level of hypothalamus ghrelin inhibits the axis, while at the level of anterior pituitary it seems to stimulate the release of LH under *in vitro* conditions [72]. Interestingly, ghrelin treatment did not change the firing and bursting activities of the GnRH neurons in proestrous mice, indicating a strong estrous-cycle dependency of the cellular processes activated by ghrelin. E2 pretreatment inhibited the ghrelin-evoked rise in the intracellular Ca^2+^-concentration in the GT1-7 neurons suggesting that E2 might be responsible for this phenomenon.

### Action of ghrelin involved activation of a retrograde endocannabinoid signaling mechanism

The results presented here revealed that the endocannabinoid signaling pathway was activated in GnRH neurons when ghrelin administration altered firing properties. Involvement of the endocannabinoid system in the function of GnRH neurons has recently been shown in our laboratory by verifying that endocannabinoids could diminish firing rate in the GnRH neuron measured [41]. Another study has also demonstrated that release of endocannabinoids could mediate the function of GnRH neurons [73]. The finding that ghrelin could trigger endocannabinoid release in GnRH neurons is supported by the above-mentioned recent work revealing that prostaglandin could also promote endocannabinoid synthesis whereas estrogen was suggested to decrease the endocannabinoid production in GnRH neurons [73].

In order to further investigate the mechanism whereby ghrelin is capable of decreasing the firing activity of GnRH neurons, whole cell patch clamp measurements were carried out. The experiments revealed that ghrelin was able to decrease the mPSC frequency. Similar effect of ghrelin has recently been described in pyramidal neurons of the lateral nucleus of the amygdala where it also diminished the frequency of mPSCs [33]. However, while the mPSCs in the amygdala related to glutamate neurotransmission [33], the mPSCs recorded in the GnRH neurons represented activation of the GABA_A_-R [41,42]. Whereas GABA acts as the major inhibitory neurotransmitter in the adult hypothalamus [74], and *in vivo* studies also showed reduced gonadotropin secretion resulted from GABA icv injection suggesting putative inhibitory effect of GABA on the GnRH neurons [75], *in vitro* experiments demonstrated excitatory responses by activating GABA_A_-R in the GnRH neurons of adult mice [29,30,76] and rats [31,77]. This contradiction can be explained by considering the different ways of application of GABA: in *in vitro* experiments GABA was applied to the GnRH neurons only, while in *in vivo* examinations GABA was administered icv and therefore it acted not only on GnRH neurons but on numerous other GABA-sensitive neurons in the close vicinity of the 3^rd^ ventricle, too [78]. The excitatory GABA neurotransmission is considered as one of the main input drives for the GnRH neurons and numerous studies demonstrated that the frequency of the GABAergic mPSCs are in a positive correlation with the firing activity of these neurons [41,79-81]. Not surprisingly, the ghrelin-evoked decrease in the firing rate of the GnRH neurons also correlated with the ghrelin-triggered reduction in the frequency of mPSCs. One can therefore conclude that the diminished firing rate observed in GnRH neurons after ghrelin administration might be partially due to the altered frequency of mPSCs resulted from application of ghrelin.

Our earlier work provided evidence that endocannabinoid release from GnRH neurons resulted in a decreased GABAergic neurotransmission to the GnRH neurons via GABA_A_-R [41]. Therefore, the ghrelin-triggered reduction in the frequency of the mPSCs found in the present experiments suggested a pivotal role of the retrograde endocannabinoid signaling machinery in the manifestation of the effect of ghrelin. Indeed, our recordings of the mPSCs of the GnRH neurons under pretreatment with AM251 or with the intracellularly applied THL showed involvement of retrograde endocannabinoid signaling mechanism in the execution of ghrelin-evoked changes observed in the mPSCs of the GnRH neuron and subsequently, in their firing. We have previously demonstrated similar ghrelin-triggered endocannabinoid machinery in other neurosecretory systems of the hypothalamus. Parvicellular neurons of the hypothalamic paraventricular nucleus, for example, were shown to synthesize and release endocannabinoids as a result of ghrelin application [32]. In the parvicellular system, the ghrelin-activated retrograde endocannabinoid signal also resulted in a decrease in the frequency of the mPSCs supporting the concept that ghrelin utilizes this machinery to alter the mPSC and firing parameters of GnRH neurons. The schematic diagram (Figure **7** **.**) describes the proposed mechanism of action of ghrelin where binding of ghrelin to GHS-R activates synthesis and release of 2-AG which then binds to CB1 located in the presynaptic terminal and eventually causing suppression of GABA release into the synaptic cleft.

It is an intriguing question, how ghrelin can access GnRH neurons, in particularly how the hormone can pass the blood-brain-barrier (BBB) to reach these neurons. Ghrelin can enter the brain via multiple routes and access the neurons rapidly. Ghrelin can leave the bloodstream at fenestrated capillaries [82]. Therefore, it is possible that it can reach the dendrites of GnRH neurons approaching the OVLT since these neurons extend complex dendritic trees outside the BBB [83]. In addition, ghrelin can pass the intact BBB utilising a triglyceride-dependent active transport system to regulate not only hypothalamic functions but learning and memory in the hippocampus [8,84,85].

The effects of ghrelin observed in our experiments resulted from the administration of acylated form of ghrelin and its binding to GHS-R expressed in GnRH neurons. This form was applied because acylation is indispensable for the binding of ghrelin to GHS-R [86]. We have to note, however, that we cannot exclude similar influence of various functions of GnRH neurons by unacylated form of ghrelin, whose concentration in plasma and circulating half-life exceeds that of acylated ghrelin. *In vivo* experiments demonstrated that unacylated form could mimic the inhibitory effects of the acylated form on the gonadal axis [87]. Both forms were able to reduce circulating level of LH and FSH under various circumstances and therefore modulating functions of gonads [87]. Unacylated form, however, is suggested to act partially via a GHS-R-independent pathway [87,88]. Therefore, putative effect of the unacylated form on the functions of GnRH neurons requires further elucidation.

In this study, we provided evidence that ghrelin is able to act directly on GnRH neurons in an estrous-cycle-dependent manner, and this action is mediated by a retrograde endocannabinoid signaling mechanism. Nevertheless, increasing amount of evidence suggests that this machinery could be involved in certain human (patho)physiological condition, too. Literature data showed that ghrelin could partially be involved in the suppression of spontaneous secretion of LH in humans [22-24]. In pathological condition, such as anorexia nervosa where high serum ghrelin level and infertility are detected, a relation between these two features of the disorder could also be hypothesized [89-93]. Nevertheless, whether such a relationship exists indeed in humans, requires further elaboration.
